# Multi-faceted approach to decreasing inappropriate antibiotic prescribing for viral upper respiratory tract infections

**DOI:** 10.1017/ash.2023.210

**Published:** 2023-09-29

**Authors:** Jamilah Shubeilat, Dan Ilges, Angie Ton, Angela Huang, M. Teresa Seville

## Abstract

**Background:** Prescribing of antibiotics for viral upper respiratory infection (URI) remains a pressing public health problem. We sought to reduce inappropriate prescribing of antibiotics for viral URIs at primary-care practices at Mayo Clinic Arizona (MCA). **Methods:** Diagnostic codes for URIs commonly caused by viruses were categorized as tier 3 (ie, never prescribe). The inappropriate prescribing rate was defined as the number of tier 3 encounters resulting in a prescription for a URI antibiotic divided by the total number of tier 3 encounters. MCA primary-care departments, including family medicine, community internal medicine, emergency medicine, and women’s health internal medicine, were included in the intervention. Each department was briefed on the project, including baseline department prescribing data, and was provided education. Education topics included appropriate indications for antibiotics, patient-centered strategies for reducing antibiotic use, and a review of electronic resources developed specifically for the project. Resources included a syndromic ambulatory order panel (EZ ID Respiratory Order Panel) and a viral prescription pad, which contains simplified over-the-counter recommendations for symptomatic management of viral URIs and patient education. Quarterly peer comparison reports were provided to the department chairs and/or site leads. Our goal was to reduce inappropriate prescribing by 22% in 2022. An Epic dashboard (SlicerDicer model) was developed to track data on an ongoing basis. We used χ^2^ tests to compare categorical variables. **Results:** Department education was completed by June 2022 (Fig. 1). The annual antibiotic prescribing rate for tier 3 encounters decreased by 29% from a baseline rate 23.6% in 2021 to 16.4% in 2022 (*P* < .001). The posteducation prescribing rate (June 2022–December 2022) was 13.1%. Utilization of the EZ ID ambulatory order panel increased from an average of 1.5 uses per month in 2021 to 13.3 uses per month in 2022 (Fig. 2). Repeated healthcare contact for URIs within 14 days of tier 3 encounters did not differ among patients prescribed and not prescribed an antibiotic in all of 2022 (3.8% vs 3.9%; *P* = .91) or during the posteducation period (1.8% vs 4.2%; *P* = .14). There was no appreciable diagnostic shift over the course of 2022 (Fig. 3). **Conclusions:** A multifaceted intervention, which included baseline education, promotion of syndrome-specific order panels, dissemination of resources for symptomatic management, and distribution of peer comparison reports, resulted in significant reduction of inappropriate antibiotic prescribing for URIs.

**Figure:**
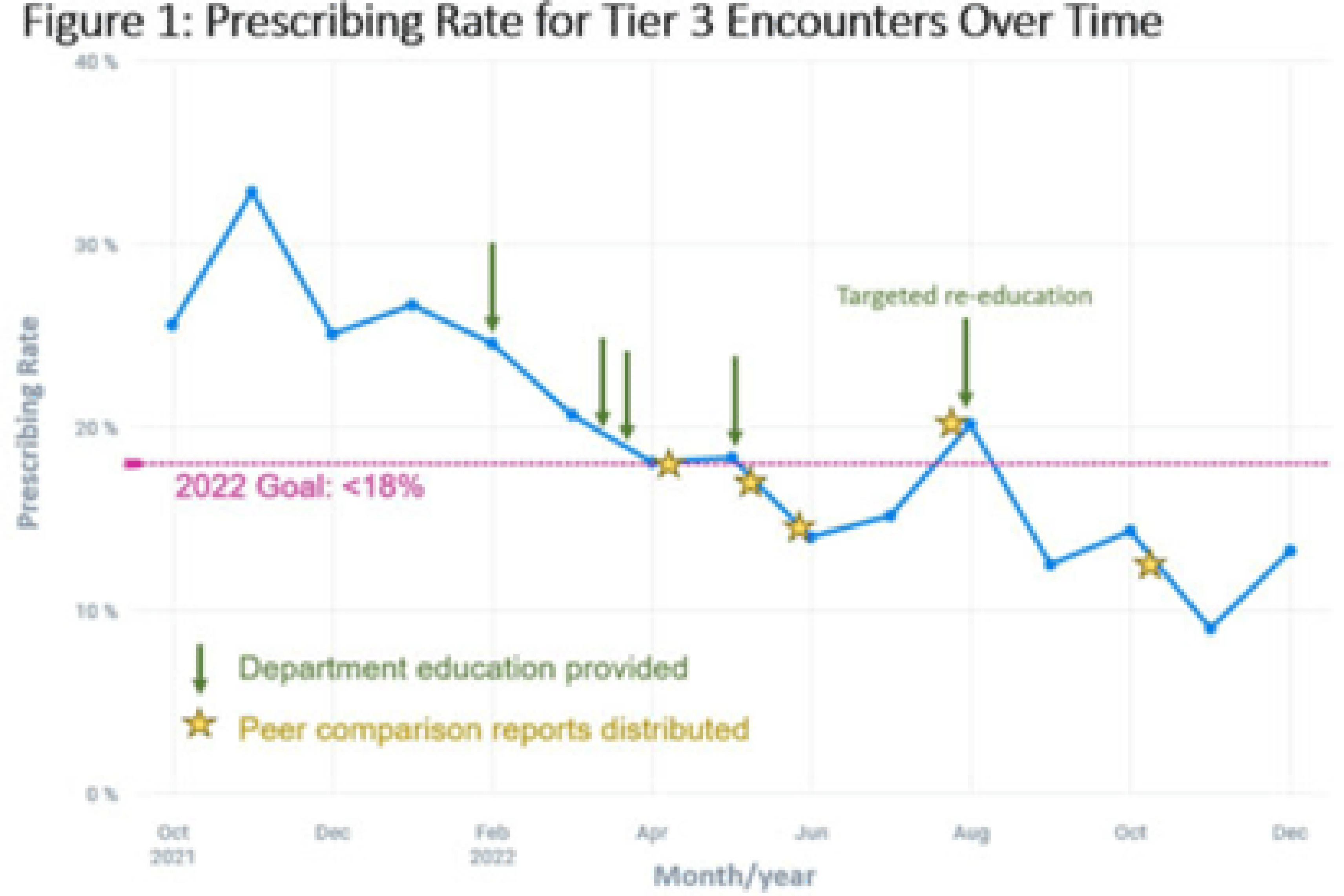

**Figure:**
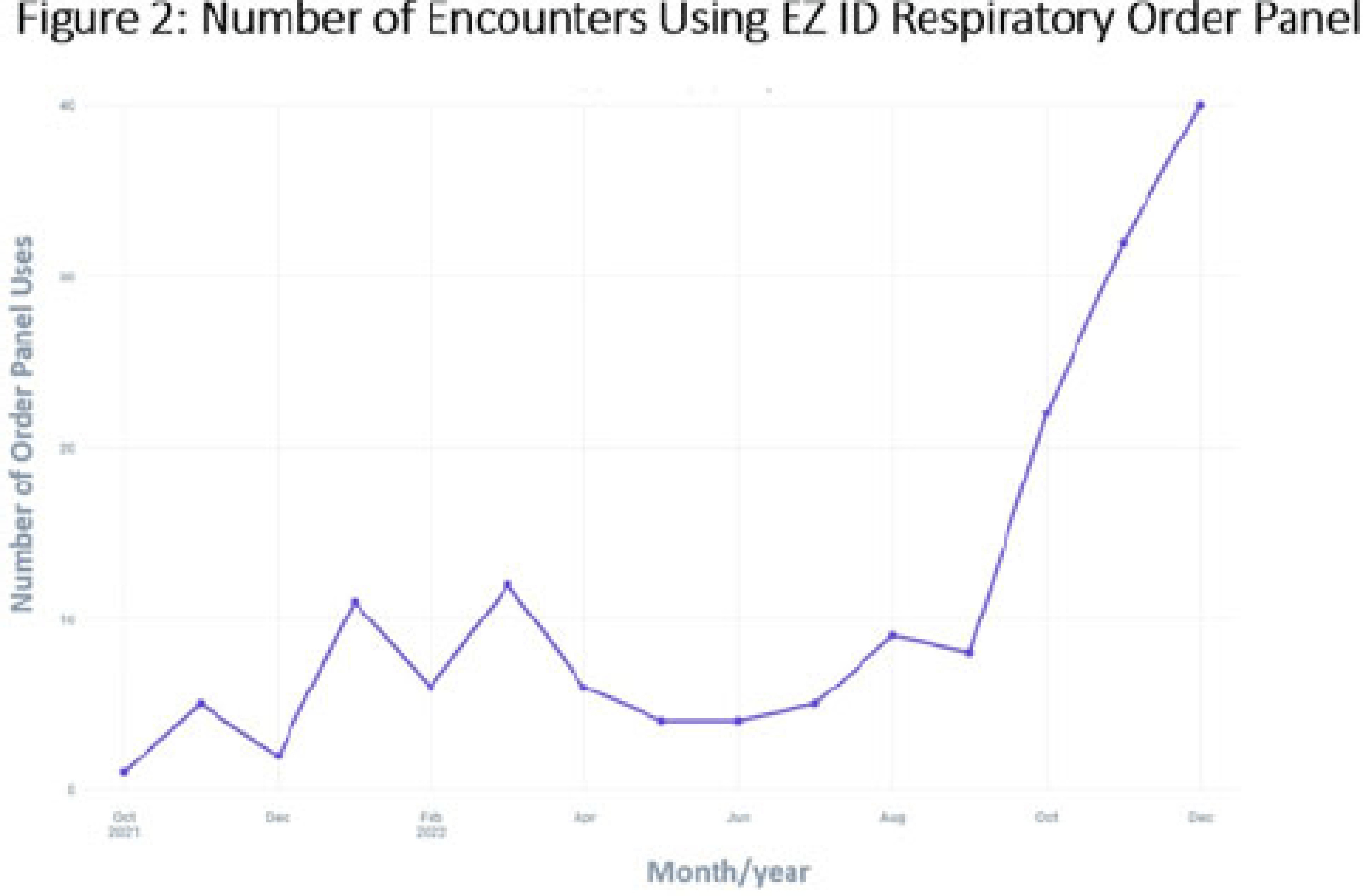

**Disclosure:** None

